# Standard Audiograms for Koreans Derived through Hierarchical Clustering Using Data from the Korean National Health and Nutrition Examination Survey 2009–2012

**DOI:** 10.1038/s41598-019-40300-7

**Published:** 2019-03-06

**Authors:** Young-Soo Chang, Sung Hoon Yoon, Jin Ryoul Kim, Sun-Young Baek, Young Sang Cho, Sung Hwa Hong, Seonwoo Kim, Il Joon Moon

**Affiliations:** 1 Department of Otorhinolaryngology - Head and Neck Surgery, Korea University College of Medicine, Korea University Ansan Hospital, Ansan, Republic of Korea; 2 Department of Otorhinolaryngology-Head and Neck Surgery, Samsung Medical Center, Sungkyunkwan University School of Medicine, Seoul, Korea; 3 Statistics and Data Center, Research Institute for Future Medicine, Samsung Medical Center, Seoul, Korea; 4 Department of Otorhinolaryngology-Head and Neck Surgery, Samsung Changwon Hospital, Sungkyunkwan University School of Medicine, Seoul, Korea

## Abstract

Assessments of standardized region/population-specific audiological characteristics are needed for provision of effective rehabilitative services through reducing costs associated with hearing aids. This study aims to propose a set of standard audiograms representing the Korean population that were derived by analyzing data from the 2009–2012 Korea National Health and Nutrition Examination Survey (KNHANES), a nationwide epidemiologic study conducted by Korean government organizations. Standard audiograms were derived by applying a hierarchical clustering method from recorded audiologic data that were obtained independently at 6 frequencies for each ear: 0.5, 1.0, 2.0, 3.0, 4.0, and 6.0 kHz (in dB HL). To derive the optimal number of clusters of the desired standard audiograms, cubic clustering criterion, pseudo-F-, and pseudo-t2-statistics were calculated. These analyses resulted in 29 clusters representing a standard audiogram of the South Korean population. Eighteen of the clusters represented normal hearing audiograms (73.11%), while 11 represented hearing-impaired (HI) standard audiograms (27.89%). Of the 11 HI audiograms, 7 were defined as flat-type (17.81%), while the remaining 4 were defined as sloping-type (9.08%). In conclusion, 29 audiograms representing standard audiograms for the Korean population have been derived using KNHANES data. Improved understanding of the characteristics of each cluster may be helpful for development of more personalized, fixed-setting hearing aids.

## Introduction

According to the World Health Organization (WHO), as of 2012, there were 360 million people in the world with hearing loss, with prevalence depending on sex, age, income, and region^1^. According to region, South Asia has the highest prevalence (27%) and the Middle East and North America the lowest (3%). Some low-income regions have twice the prevalence of hearing loss of high-income regions (11%). Population origin/ethnicity also plays a role in the prevalence of hearing loss^2^.

Despite the growing number of people with hearing impairment, the hearing aid adoption rate is staggeringly low, at only 1 in 5 hearing-impaired people. There are several reasons for this low adoption rate, but one of the main reasons is the high cost of the devices^3,4^. Hearing aid ownership is the lowest among socioeconomically disadvantaged groups, including ethnic minorities and those with the lowest levels of income and education^5^. Currently, hearing aids are fitted by experienced health care professionals, and this fitting procedure increases the price of hearing aids. Self-fitting hearing aids, personal sound amplification products (PSAP), or devices with several fixed fitting modes are now available to reduce costs. Simple, useful amplification formulae are necessary for these devices to be used universally.

Before providing formalized amplification formulas, it may be beneficial to address the nature of “standard audiograms.” The use of standard audiograms for hearing aid design was first suggested by the Nordic Cooperation on Disability (NSH) in 2003, during discussions about modernizing hearing aid measurement standards. The proposed sets of audiograms are intended to be used for hearing aid measurements in which the effects of fitting or the use of certain features, such as wireless streaming technology or noise reduction technology, must be demonstrated objectively. This information would be used to generate data and instruction for use on hearing aids, hearing aid features, and fitting methods.

Several studies have established standardized audiograms. In 2003, the NSH first proposed a set of five audiograms, representing (1) mild sensorineural loss, (2) moderate sensorineural loss, (3) severe sensorineural loss, (4) profound sensorineural loss, and (5) precipitous sensorineural loss, purely based on their experience^6^. However, the proposed audiograms accounted only for 26% of patients when checked against a database of 15,000 standard audiograms from the Stockholm South Hospital. In 2010, the International Standards for Measuring Advanced Digital Hearing Aids (ISMADHA) group proposed a set of 10 standard audiograms using a statistical approach that applied to 46% of the 28,244 audiograms used in the study^6^. In the context of such variation, it could be beneficial to provide standard audiograms representing region/population-specific hearing loss trends. Assessment of standardized region/population-specific audiological characteristics is needed by healthcare providers seeking to create effective rehabilitative services^7^. To our knowledge, there are no studies of hearing impairment trends in East Asia, and standard audiograms based on data from nationwide epidemiologic studies set in East Asia are lacking.

This study aims to propose a set of standard audiograms representing Koreans generated through hierarchical clustering analysis of data from the 2009–2012 Korea National Health and Nutrition Examination Survey (KNHANES), a nationwide epidemiologic study conducted by Korean government organizations.

## Methods

The study used a statistical approach to create standardized audiograms through hierarchical clustering analysis to represent trends of hearing loss in the South Korean population. Written informed consent was obtained from all participants before the survey, and approval for this research was obtained from the Institutional Review Board of Samsung Medical Center (IRB No. 2013-02-031).

### KNHANES

KNHANES is a nationwide survey that is performed annually by the Korea Centers for Disease Control and Prevention to analyze the health and nutritional statuses of a representative Korean population sample. This survey is a cross-sectional survey of the civilian population aged ≥1 year living in households in South Korea and is described in detail elsewhere^8–10^. In the KNHANES, a field survey team consisting of an otolaryngologist and nurse performs interviews and physical examinations. Selected participants undergo basic otolaryngologic examinations. A history of otological symptoms is surveyed, and physical examinations including the tympanic membrane, hearing, and balance along with pure tone audiometry are conducted in participants of appropriate ages. Every year, 10,000–12,000 people in approximately 3,800 households are selected from a panel to represent the Korean population using a multistage clustered and stratified random sampling method based on Korean National Census Data. From the chosen data set, 192 survey sections and 20 households were selected from each section. The participation rates for the medical examinations were high: 79.2%, 77.5%, 76.1%, and 75.9% for 2009–2012, respectively.

### Audiometric measure

Pure tone audiograms were measured by a trained otolaryngologist using an automatic audiometer (GSI SA-203; Entomed Diagnostics AB, Lena Nodin, Sweden) in a soundproof booth. Pure tone thresholds were obtained independently at 6 frequencies for each ear: 0.5, 1.0, 2.0, 3.0, 4.0, and 6.0 kHz. The pure-tone average (PTA) was obtained as the average threshold at 0.5, 1.0, 2.0, and 4.0 kHz. To ensure reliability, the Epidemiologic Survey Committee of the Korean Society of Otorhinolaryngology-Head and Neck Surgery periodically organized seminars for otolaryngologists as a means of quality control.

### Inclusion and exclusion criteria

Pure tone audiograms were conducted in participants aged 12 years or older who were eligible for the survey. Out of the 34,251 participants who took part in the 2009–2012 KNHANES, 18,415 (≥12 years) underwent medical examinations including pure tone audiometry. Audiograms from 36,828 ears were selected after excluding missing and erroneous measurements. Each individual participant represented the Korean population at a different weight calculated by the sampling rate, response rate, and age/sex proportions of the reference population^11^. Using sample weight, this survey provided representative estimates of the Korean civilian population living in households. Audiogram data ([subjects/households/survey areas/year] = u[10,078/4,600/200/2009], [8,473/3,840/192/2010], [8,055/3,840/192/2011], [7,645/3,840/192/2012], respectively) were obtained from the 2009–2012 KNHANES. Such data can be powerful tools for investigating the national prevalences of specific diseases and health behaviors. KNHANES data were used to produce a standard audiogram to comprehensively represent hearing impairment trends in Korea.

### Statistical analysis

Standard audiograms were obtained by applying a hierarchical clustering method to derive new standard audiograms from the total data set of recorded audiograms showing hearing thresholds (dB HL) at six frequencies of 500, 1,000, 2,000, 3,000, 4,000, and 6,000 Hz. Hierarchical clustering is a method of cluster analysis that seeks to build a hierarchy of clusters. To derive the optimal number of clusters of desired standard audiograms, cubic clustering criterion (CCC)^12^, pseudo-F-, and pseudo-t2-statistic^13^ were calculated. The CCC can be used to estimate the number of clusters using Ward’s minimum variance method, k-means, or other methods based on minimized within-cluster sum of squares. Statistical analyses were executed using SAS version 9.4 (SAS Institute, Cary, NC, USA).

The suggested audiograms were categorized into two groups according to PTA: 1) normal hearing (NH) audiogram if the PTA was below 25 dB and 2) hearing-impaired (HI) audiogram if the PTA was equal to or greater than 25 dB. If the difference between two adjacent frequencies was equal to or greater than 20 dB, they were defined as steeply sloping losses.

## Results

**Figure 1:**
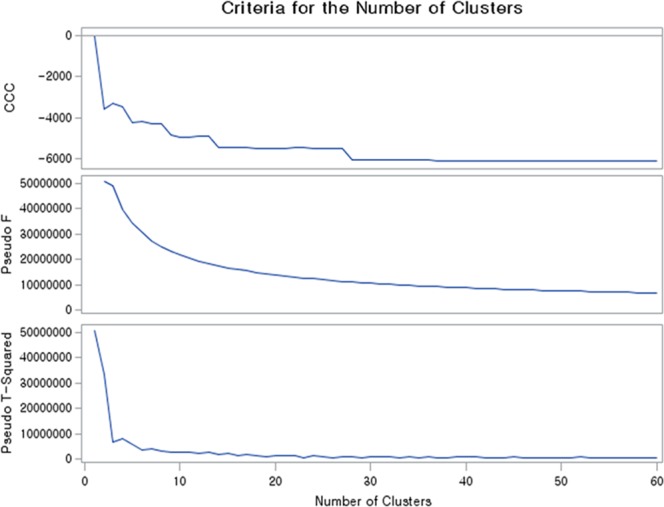
Criteria for selecting the number of clusters. (**a**) Cubic clustering criterion (CCC), (**b**) pseudo-F-statistic, and (**c**) pseudo-t2-statistic.

**Figure 2:**
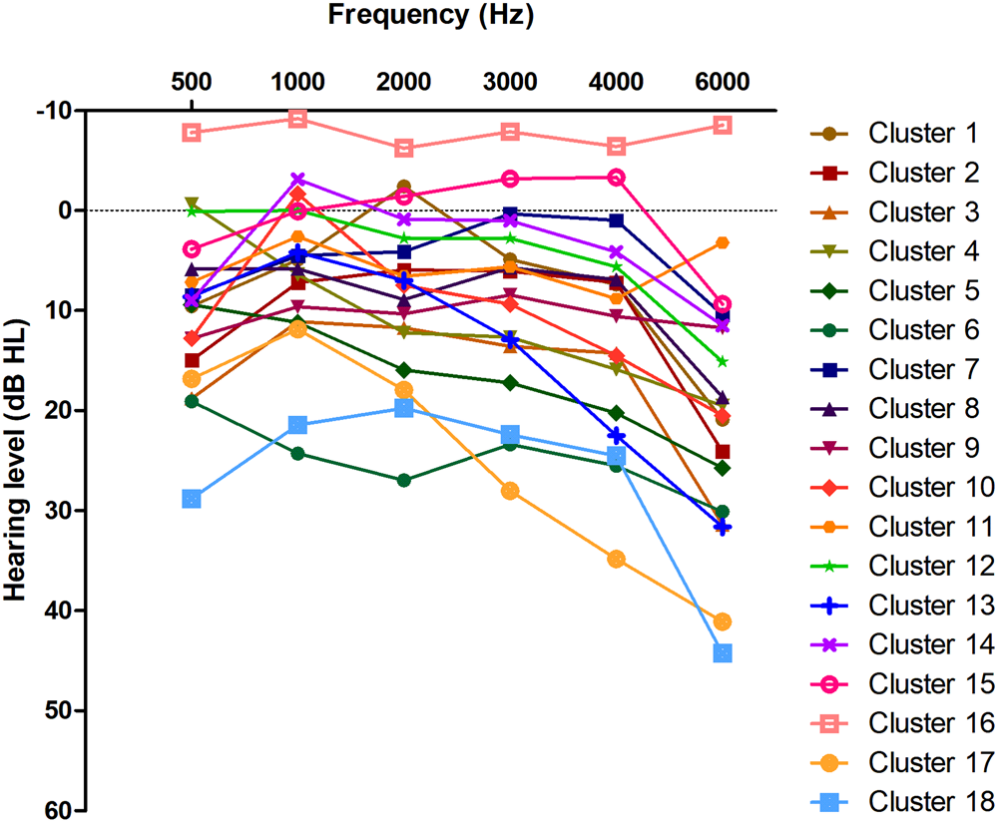
Proposed normal hearing standard audiograms.

The statistical method resulted in 29 clusters representing the standard audiogram of Korea. Figure 1 summarizes the results derived from hierarchical clustering with CCC, pseudo-F-, and pseudo-t2-statistic for optimal cluster number detection. Among the 29 representative standard clusters, 18 represented NH audiograms, and 11 represented HI standard audiograms. The NH audiograms account for 73.11% of audiograms, and HI audiograms account for 27.89%. The detailed hearing thresholds at each frequency in each cluster are summarized in Table 1. The overall standard NH audiograms are shown in Fig. 2.

**Table 1:** Standard audiograms.

Cluster	Type	Percentage (%)	500 Hz	1000 Hz	2000 Hz	3000 Hz	4000 Hz	6000 Hz	Average PTA
1	Normal	3.25	9.56	4.92	−2.4	4.9	7.4	20.89	4.87
2		6.71	14.97	7.2	5.93	6.05	7.18	24.1	8.82
3		6.91	18.8	11.09	11.72	13.58	14.25	31.38	13.965
4		1.92	−0.64	6.4	12.22	12.66	15.88	19.5	8.465
5		6.5	9.43	11.18	15.95	17.21	20.24	25.75	14.2
6		3.09	19.07	24.29	26.97	23.39	25.52	30.1	23.9625
7		6.75	8.49	4.52	4.11	0.32	0.99	10.4	4.5275
8		4.04	5.86	5.82	8.9	5.72	6.92	18.65	6.875
9		6.19	12.82	9.62	10.33	8.44	10.58	11.73	10.8375
10		1.8	12.78	−1.67	7.49	9.37	14.51	20.51	8.2775
11		2.25	7.16	2.62	6.56	5.64	8.82	3.21	6.29
12		3.77	0.11	−0.02	2.79	2.79	5.62	15.1	2.125
13		5.03	8.63	4.16	7	12.88	22.5	31.6	10.5725
14		2.29	8.98	−3.11	0.88	1	4.14	11.5	2.7225
15		3.77	3.86	0.07	−1.42	−3.18	−3.31	9.37	−0.2
16		0.17	−7.79	−9.18	−6.23	−7.87	−6.39	−8.52	−7.3975
17		4.75	16.83	11.87	17.93	28.01	34.83	41.11	20.365
18		3.92	28.79	21.45	19.76	22.4	24.51	44.27	23.6275
19	HI-Flat	1.44	90.17	89.71	91.3	93.79	95.07	101.87	91.56
20		2.67	51.07	53.38	61.05	69.37	72.94	86.26	59.61
21		3.36	34.52	36.44	44.79	56.42	61.9	75.2	44.41
22		4.31	16.52	19.24	26.59	33.63	41.95	59.57	26.08
23		2.94	31.08	30.16	32.83	40.63	46.89	65.43	35.24
24		1.16	51.47	41.14	33.74	34.51	35.26	50.11	40.40
25		1.93	6.66	4.99	9.68	29.18	44.27	40.48	16.4
26	HI-Sloping	3.63	17.06	21.18	42.54	56.08	61	69.63	35.45
27		1.65	11.4	11.14	12.74	13.82	20.07	56.78	13.84
28		2.13	13.05	10.75	19.99	55.58	68.91	74	28.18
29		1.67	14.35	7.01	9.89	23.19	50.63	65.18	20.47

HI, hearing-impaired; PTA, pure-tone averages at 500, 1000, 2000, and 4000 Hz.

**Figure 3:**
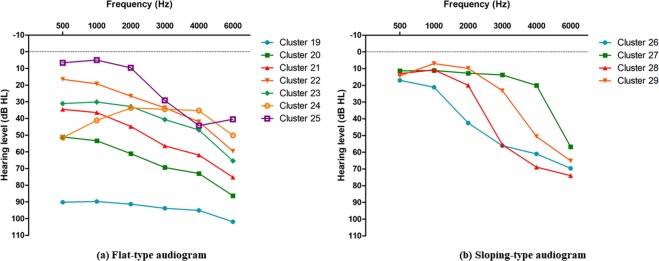
Proposed hearing-impaired standard audiograms. (**a**) Flat-type audiogram and (**b**) sloping-type audiogram.

Of the 11 HI audiograms, 7 can be defined as flat-type, (17.81%), and 4 can be defined as sloping-type (9.08%) (Fig. 3). Sex and age distributions among the standard HI audiograms are summarized in Tables 2 and 3, respectively. Flat-type HI audiograms showed the most common age band in the 61–80 year range except for clusters 24 and 25, which exhibited a female predominance and male predominance, respectively. In addition, cluster 24 (1.16% of the overall population) showed a flat HI audiogram. However, this audiogram showed a rising pattern from 500 and 1000 Hz to 2000 Hz. In cluster 25 (1.83% of the overall population), a C-5 (4000 Hz) dip was observed.

**Table 2:** Sex distribution among the standard hearing-impaired audiograms.

Cluster	Type	Male				Female			
		Frequency	Percentage (%)	Weighted frequency	Weighted percentage (%)	Frequency	Percentage (%)	Weighted frequency	Weighted percentage (%)
19	Flat	220	41.51	338,803	43.63	310	58.49	437,780	56.37
20		521	52.89	712,757	52.72	464	47.11	639,223	47.28
21		640	51.65	952,236	54.83	599	48.35	784,447	45.17
22		779	49.09	1,339,050	55.59	808	50.91	1,069,587	44.41
23		400	36.93	627,460	39.46	683	63.07	962,689	60.54
24		102	23.94	193,872	26.73	324	76.06	531,520	73.27
25		580	81.81	1,402,586	86.45	129	18.19	219,753	13.55
26	Sloping	930	69.51	1,460,170	72.25	408	30.49	560,797	27.75
27		298	48.93	717,549	58.89	311	51.07	500,876	41.11
28		714	91.19	1,385,499	93.66	69	8.81	93,742	6.34
29		496	80.65	1,054,668	84.78	119	19.35	189,270	15.22

**Table 3:** Age distribution among standard hearing-impaired audiograms.

Cluster	Type	Weighted percentage (%) of each age band						
		~30	31–40	41–50	51–60	61–70	71–80	81~
19	Flat	4.62	10.65	12.05	19.15	19.22	*25*.*60*	8.72
20		2.29	2.53	6.81	12.82	27.51	*37*.*12*	10.92
21		0.91	2.08	5.98	15.17	29.24	*36*.*84*	9.77
22		1.40	4.39	11.88	27.94	*30*.*23*	21.72	2.44
23		0.97	2.35	7.56	17.98	30.06	*34*.*54*	6.54
24		7.53	8.07	17.43	*23*.*59*	22.14	18.42	2.79
25		4.99	16.92	*39*.*20*	25.04	11.12	2.70	0.04
26	Sloping	0.98	3.53	8.77	26.32	*31*.*06*	25.12	4.22
27		8.00	14.09	*32*.*29*	25.04	15.34	5.16	0.07
28		1.02	8.18	29.46	*31*.*23*	20.66	9.07	0.37
29		4.05	14.51	*30*.*98*	28.55	13.16	8.62	0.12

In contrast to the flat-type HI audiograms, the peak age band of the sloping type HI audiogram lies in the 41–60 year range except for cluster 16, which represents gradual hearing loss with a steep change between 1000 and 2000 Hz. Clusters 27, 28, and 29 show normal hearing thresholds below the frequency of steep change (cluster 27: 6000 Hz, cluster 28: 3000 Hz, cluster 29: 4000 Hz).

## Discussion

This is the first study to propose standard audiograms based on nationwide epidemiologic study data. The present study is based on the 2009–2012 KNHANES, which provides powerful tools for investigating the national prevalences of specific diseases and health behaviors. The 29 proposed standard audiograms represent the national HL trends of South Korea, rather than encompassing only a city and its surrounding suburbs. Moreover, since only ethnically Korean individuals participated in the KNHANES, the proposed standard audiograms are valuable to compare HL trends between populations, especially since there are abundant data regarding the hearing trends of Europeans, African-Americans, and Hispanics (Lin *et al*.^2^).

A total of 29 clusters representing hearing trends in South Korea were acquired via hierarchical clustering analysis using the 2009–2012 KNHANES database. Excluding 18 clusters that represented NH trends, further analyses were performed on the remaining 11 clusters representing HL trends in Korea.

The 11 proposed standard audiograms were separated into sloping standard audiograms or flat standard audiograms depending on steepness. Each audiogram infers clinical information. Cluster 19 accounts for 1.44% of the overall data when each ear was evaluated separately. Although the contralateral ear hearing thresholds were unknown, this cluster indicates a group that may need cochlear implants for hearing rehabilitation. Clusters 20, 21, 23, and 24 account for 10.13% of the overall data and represent good candidates for hearing aid rehabilitation. Clusters 20, 21, and 23 showed a higher age distribution of 71–80 years, at which hearing aids are usually required. Therefore, these are appropriate clusters for preparing standardized hearing-aid fitting formulae.

Interestingly, cluster 24 represents low-frequency hearing loss and is associated with females and an age-distribution of 51–60 years. This may reflect the so-called sex-reversal phenomenon, reported in many studies^14^, which suggests that elderly women have slightly poorer low-frequency hearing than men of similar age.

Cluster 25 showed male predominance, C-5 dip, and an older age distribution of 41–50 years. In addition to cluster 25, clusters 27 and 29 also suggest high-frequency hearing loss with male predominance. These standard audiograms may be associated with male whose working environment is very noisy^15,16^. Since every Korean male has an obligation to participate in military service, these audiograms may also be associated with previous exposure to intense sounds during military drills^17,18^. Using hierarchical clustering analysis, the relevant interest group can be identified and analyzed in future studies.

Cluster 22 represents gradual slopping type hearing loss and accounts for 4.31% of the overall data. This cluster ranges from 51–80 years and showed male predominance. This cluster may infer age-related hearing loss, similar to the results of several epidemiological studies of middle-aged and elderly people indicating that males have more high-frequency hearing loss than females^19–21^. Clusters 26 and 28 account for 5.76% of the overall data. These groups may be experiencing hearing discomfort, according to the contralateral hearing threshold. However, appropriate hearing aid fitting could be difficult due to occlusion effects associated with good low-frequency hearing thresholds.

Currently, individuals with hearing loss can buy PSAP or over–the–counter (OTC) hearing aids. Expert intervention during the hearing aid fitting process is reduced with the use of these devices, and self-fitting will be more widely performed in the future. Standard audiograms are beneficial to help develop formalized amplifications for self-fitting, which can be operationalized as preset modes. Such features reduce the cost of hearing rehabilitation and are helpful for improving the experience when people with hearing loss begin to use PSAP or OTC hearing aids. The present study results provide a range of seven flat and four sloping audiograms that are applicable to hearing-impaired populations in Korea. Identifying representative audiograms is helpful to produce standardized products such as PSAPs or basic HAs to provide users with more personalized, fixed settings. Fixed settings that are appropriate for different regions or ethnicities and that are based on standard audiograms offer much higher likelihood of achieving optimal fit using fixed settings provided for a reasonable price.

This study has some limitations. Although the clustering analysis incorporated a large set of nationwide data, and the results may be referred to as standard audiograms, causal or associated factors of hearing loss were not evaluated, and it is difficult to explain the characteristics of each standard audiogram. This may restrict the understanding and use of standard audiograms. Second, while the pure tone audiograms were measured by a trained otolaryngologist with an automatic audiometer, bone-conduction hearing thresholds were not determined. If bone-conduction hearing thresholds were available, those data may be helpful to suggest more optimal treatments for each patient group. However, hearing thresholds obtained from subjects with normal tympanic membranes were used in this study, and this could minimize the bias through which conductive hearing loss affects clustering outcomes.

## Conclusion

Twenty-nine audiograms representing the population of South Korea were proposed using KNHANES data. This is the first study to propose standard audiograms based on nationwide epidemiologic study data. The results suggest 18 clusters representing normal hearing trends and 11 clusters representing hearing loss trends in South Korea. A greater understanding of the characteristics of each cluster would be helpful for development of more personalized fixed-setting HAs such as PSAPs or basic HAs. These would lower costs and make HAs accessible to more users.

## References

[1] World Health Organization. WHO global estimates on prevalence of hearing loss. *Geneva: World Health Organization* (2012).

[2] Lin FR (2012). Association of skin color, race/ethnicity, and hearing loss among adults in the USA. Journal of the Association for Research in Otolaryngology.

[3] Yoon SH (2017). A Trainable Hearing Aid Algorithm Reflecting Individual Preferences for Degree of Noise-Suppression, Input Sound Level, and Listening Situation. Clin. Exp. Otorhinolaryngol..

[4] Fischer ME (2011). Determinants of hearing aid acquisition in older adults. Am. J. Public Health.

[5] Bainbridge KE, Ramachandran V (2014). Hearing aid use among older U.S. adults; the national health and nutrition examination survey, 2005–2006 and 2009–2010. Ear Hear..

[6] Bisgaard N, Vlaming MS, Dahlquist M (2010). Standard audiograms for the IEC 60118-15 measurement procedure. Trends in amplification.

[7] Blustein J, Weinstein BE (2016). Opening the Market for Lower Cost Hearing Aids: Regulatory Change Can Improve the Health of Older Americans. Am. J. Public Health.

[8] Cho Y-S (2010). Prevalence of otolaryngologic diseases in South Korea: data from the Korea national health and nutrition examination survey 2008. Clin. Exp. Otorhinolaryngol..

[9] Chang Y-S, Choi JE, Kim SW, Baek S-Y, Cho Y-S (2016). Prevalence and associated factors of facial palsy and lifestyle characteristics: data from the Korean National Health and Nutrition Examination Survey 2010–2012. BMJ open.

[10] Park KH (2014). Prevalence and associated factors of tinnitus: data from the Korean National Health and Nutrition Examination Survey 2009–2011. J. Epidemiol..

[11] Kweon S (2014). Data Resource Profile: The Korea National Health and Nutrition Examination Survey (KNHANES). Int. J. Epidemiol..

[12] Sarle, W. S. & Institute, S. *Cubic Clustering Criterion*. (SAS Institute, 1983).

[13] Institute, S. *SAS/STAT user’s guide: version 6*. Vol. 2 (Sas Inst, 1990).

[14] Jerger J, Chmiel R, Stach B, Spretnjak M (1993). Gender affects audiometric shape in presbyacusis. J. Am. Acad. Audiol..

[15] Consensus conference. Noise and hearing loss. *JAMA* **263**, 3185–3190 (1990). 2190006

[16] Nandi SS, Dhatrak SV (2008). Occupational noise-induced hearing loss in India. Indian J. Occup. Environ. Med..

[17] Ryan AF, Kujawa SG, Hammill T, Le Prell C, Kil J (2016). Temporary and Permanent Noise-induced Threshold Shifts: A Review of Basic and Clinical Observations. Otol. Neurotol..

[18] Chang Y-S, Bang KH, Jeong B, Lee G-G (2017). Effects of early intratympanic steroid injection in patients with acoustic trauma caused by gunshot noise. Acta Otolaryngol..

[19] Cruickshanks KJ (1998). Prevalence of hearing loss in older adults in Beaver Dam, Wisconsin: The epidemiology of hearing loss study. Am. J. Epidemiol..

[20] Gates GA, Cooper JJ, Kannel WB, Miller NJ (1990). Hearing in the elderly: the Framingham cohort, 1983-1985. Part I. Basic audiometric test results. Ear Hear..

[21] Jönsson R, Rosenhall U, Gause-Nilsson I, Steen B (1998). Auditory function in 70-and 75-year-olds of four age cohorts. Scand. Audiol..

